# Nationwide, Multioperator Self-Exclusion and Psychiatric Comorbidity in Patients with Gambling Disorder: A Retrospective Chart Review Study from a Regional Treatment Unit

**DOI:** 10.1155/2023/5532259

**Published:** 2023-09-28

**Authors:** M. Miles, J. Rothschild, G. Åkesson, A. Håkansson

**Affiliations:** ^1^Lund University, Faculty of Medicine, Lund, Sweden; ^2^Bryta Punkt Nu, Malmö, Sweden; ^3^Lund University, Department of Clinical Sciences Lund, Division of Psychiatry, Lund, Sweden

## Abstract

Gambling disorder is an addictive disorder that has been shown to have a detrimental effect on an individual's health, social, and financial situations. Voluntary self-exclusion is one way for patients to reduce harm in gambling disorder, but breaching one's self-exclusion appears to be common. In January 2019, Sweden launched a nationwide, multioperator self-exclusion instrument called *Spelpaus* (literally “game break”). *Spelpaus* is unique to Sweden, and there is limited research on the use of this type of nationwide, multioperator self-exclusion services, also in relation to gambling disorder and mental health. There is a reason to follow the clinical picture of treatment seeking for gambling disorder over time, and this study aims to explore clinical characteristics of patients seeking clinical gambling disorder treatment, including sex distribution and mental health comorbidity, as well as the use of *Spelpaus* amongst patients with gambling disorder and how frequently users gambled despite ongoing self-exclusion, in relation to sex and psychiatric comorbidities. A retrospective chart study was carried out on patients presenting to a regional gambling disorder treatment unit. Information regarding self-exclusions using *Spelpaus*, gambling despite self-exclusion, and the method of gambling despite self-exclusion as well as psychiatric comorbidities were extracted from medical records. Females were markedly more likely to report overall psychiatric comorbidities (48% vs. 25% among males, *p* < 0.001), affective, neurotic/anxiety-related (*p* < 0.001), and behavioral/emotional (*p* = 0.028) diagnoses and more likely to have two or more diagnoses excluding gambling disorder (*p* = 0.001). From 120 patients from whom information regarding self-exclusion was present, 114 (95%) had chosen to self-exclude. From the 114 self-excluders, 67 reported to have gambled despite self-exclusion, with unregistered websites being the most common method. Self-exclusion was not significantly related to sex (*p* = 0.146) or to psychiatric comorbidities (*p* = 0.178). In conclusion, psychiatric comorbidity was particularly common in female gambling disorder patients and gambling despite self-exclusion was common. Gambling regulations should be improved to help self-excluders avoid being able to gamble on unlicensed gambling operators. Further research should focus on sex differences and the association with psychiatric comorbidities.

## 1. Introduction

Gambling disorder is defined by the fifth edition of *Diagnostic and Statistical Manual* (DSM-5) as an addictive disorder, which is characterized by its impairing effects on the individual's mental health and financial situation [1]. The lifetime prevalence of problem gambling ranges between 0.12% and 5.58% in countries around the world, varying in part because of factors such as study design and diagnostic criteria. The prevalence has also shown to be slightly higher in subgroups in particular contexts, for example, in psychiatric inpatients and in those who are receiving treatment for substance use. Mental illness is markedly more common in individuals with gambling problems compared with those who do not gamble and those who do not have a problematic approach towards gambling. Demographic factors such as lower educational level, low income status, or low socioeconomic status have also been demonstrated to correlate with higher prevalence of problematic gambling [1–3].

There are described prevalence differences between the sexes, and the reported ratio is at least 2 : 1 in males compared to females [3]. Sex-related diversities also appear to occur in individuals with gambling problems. Female gamblers tend to have a higher onset age compared to the male ones, but they show a more rapid progression to problem gambling, known as a *telescoping effect*. Studies present that females are more likely to take part in nonstrategic and less interactive gambling setups, such as bingo or online slot machines, while males are more prone to choose strategic gambling types, such as blackjack or gambling related to sports [4]. Males are more often reported to have substance use disorders in combination with their gambling disorder, although females show an overall higher prevalence in getting treatment for a nongambling-related mental illness [4]. There is further a well-documented comorbidity with other psychiatric diagnoses in combination with gambling disorder, such as substance use and anxiety disorders [5–7], and also suicidal thoughts, suicide attempts [8], and completed suicides [9].

Treatment seeking in gambling disorder is typically low [10, 11], and in many settings, there is a lack of structured treatment uptake for this condition in traditional medical or social treatment settings. In the setting studied here, Sweden, the first specialized treatment unit for gambling disorder in the hospital system was opened in 2015, and some early experience from this treatment setting demonstrated high psychiatric comorbidities, clear sex differences in gambling types and psychiatric diagnoses, and a predominating role of online casino. However, treatment for GD in this and many other settings is sparse, and there is a reason to follow the clinical picture of patients seeking treatment over time [12].

Gambling disorder can today be treated with cognitive behavioral therapy or motivational interventions, but treatment is meeting difficulties [13]. The online gambling market is growing and shows distinctive features of being fast-paced and easily available, which complicates management of the condition [14]. Relatively, few with gambling problems are seeking help for their condition. This may, for example, be due to stigma, feelings of guilt, lack of support, or individuals wanting to take care of the problem on their own. Another barrier can be patients having limited access to treatment, by reason of long distance to a clinic or absence of peer support groups in the area [10]. Therefore, other methods are under development and new self-help ways, such as self-exclusion, are being tried [15].

Prior self-exclusion programmes have been in use, where a person at risk for gambling disorder or who has already developed gambling disorder has the choice to self-exclude during a period from a single casino or online gambling operator, this being a preventive measure to hinder problematic gambling. These programmes show limitations, considering they have mostly been applied in land-based gambling and casinos. Also, the programmes require the patient to self-exclude from every site separately. Accessing the gambling site/venue in order to self-exclude from that place implies a temptation to gamble and a risk to relapse. Considering the number of gambling operators, it is difficult to self-exclude from all of them, and therefore, gambling via another operator is easily accessible [16–18]. Based on the results from these studies, a period of self-exclusion has resulted in lower rates of pathological gambling at follow-up than those before self-exclusion [18].

On January 1, 2019, the Swedish Gambling Authority launched a national self-exclusion instrument called *Spelpaus* (https://www.spelpaus.se), which directly translates to “*gambling break*.” It is a unique Swedish self-exclusion service which allows individuals above the age of 18, regardless of prior gambling problems or even regardless of prior gambling experience, to voluntarily suspend from all licensed online gambling sites and land-based casinos and betting venues in Sweden. The individual can do this by visiting the independent website of *Spelpaus* and registering by using an official online identification service. Therefore, there is no need to log on to every online gambling site separately [19].

The time of self-exclusion can be chosen to be either one, three, or six months [20]. It is also possible to self-exclude for an unlimited time with the possibility to discontinue after 12 months. The individual cannot cancel an ongoing self-exclusion period, but it is possible to prolong it. Licensed gambling sites electronically check the *Spelpaus* register every time an individual registers to or logs on to the gambling site, such that gambling is only permitted if the individual is currently not self-excluded. Since the launch of *Spelpaus*, the number of individuals with ongoing self-exclusions increased to close to 80,000 in September 2022. From the 80,000, 75% were reported to be male and two-thirds had chosen to self-exclude for an unlimited amount of time [21]. Legal gambling types that *Spelpaus* does not apply to are lottery tickets purchased in grocery stores, kiosks, and similar and smaller gambling services in bars and restaurants that are limited with respect to the deposit of smaller amounts of money, commonly referred to as “restaurant casinos” [22].

In the Swedish gambling market, currently, around 80 operators hold a license to operate within the country, in most cases in only online casino and/or online sports betting services. *AB Svenska Spel* is a state-owned gambling operator providing online poker, online bingo, online casino games, online lotteries, sports betting, and land-based electronic gambling machines. A subdivision of *AB Svenska Spel* owns the three existing state-owned land-based casinos. In addition, a number of operators provide only physical or online-based lottery or scratch lottery gambling. These licensed operators are all included in the *Spelpaus* system.

As *Spelpaus* allows individuals to exclude from around 80 gambling operators with a Swedish license without accessing gambling venues or websites, *Spelpaus* is a unique instrument with few or none existing comparable tools in other countries. Considering *Spelpaus* is also a relatively new service, this study is one of the first to describe the use of a nationwide self-exclusion tool in patients with a gambling disorder.

In many countries, offline gambling venues such as land-based casinos closed temporarily due to the COVID-19 pandemic [23–27]. There were also a lot fewer sporting events as a result of the pandemic [28], decreasing the market for sports betting [29]. In a literature review from April 2021, all 17 studies from around the world showed a general overall decrease in gambling, with only a subgroup of people increasing their expenditure and time spent gambling [30]. However, in Sweden, the turnover of major gambling operators in Sweden remained unchanged during the pandemic and possibly also increased throughout the most intense parts of the pandemic [31], which has sparked the question if individuals that gambled in offline venues or were betting on sports instead turned to online alternatives and that previous decreases measured in gambling were transient decreases in the beginning of the pandemic. In Swedish legislation, a specific COVID-19-related preventive measure was introduced in July 2020, and in effect through 2021, which limited the weekly amount of money one could lose on each separate operator in online casino or electronic gaming machines (a maximum of 5,000 SEK/week, corresponding to around 450 euros/week), and also limiting the amount of free bonus possible to offer to new clients. This intervention has been discussed as possibly counteracting lifestyle changes which could potentially increase gambling behavior during the pandemic, but a fear also has been expressed that the intervention may increase nonlicensed gambling, although data hitherto have not supported this [32]. Thus, based on this, there is also a reason to assess self-exclusion and breaching of self-exclusion in the context of COVID-19 and the related government intervention, in clinical patients.

The aim of this study was to describe changes in the clinical picture of gambling disorder patients at a specialize treatment unit, following the previous early experience from the same unit [12], and with a focus on the prevalence and nature of comorbidity in patients with gambling disorder, sex differences, and its relation to voluntary self-exclusion following the introduction of the national *Spelpaus*self-exclusion service. The study specifically aimed to assess this self-exclusion service including the risk of self-reported breaching of this self-exclusion, also in relation to the COVID-19 pandemic.

Specifically, the study involved the following research questions: To what extent had patients with gambling disorder chosen to self-exclude using *Spelpaus*, based on medical records since the launch of the service? How common was gambling despite self-exclusion? What was the prevalence and nature of psychiatric comorbidity over time in patients with gambling disorder, with regards to sex differences? Considering the global COVID-19 pandemic, did the number of patients, and the rates of self-exclusion and breaching, change between 2017 and August 2021?

## 2. Methods

Malmö Addiction Center is the only medical center specialized in gambling disorder in the Skåne region, Sweden (a regional uptake area involving a population of 1.4 million inhabitants). The unit is part of the public addiction health care in the region. In Sweden, health care is either entirely public or private with public financing, such that it is available for all at a very limited cost and without involvement of private insurances or similar. Gambling is regulated in a license-based gambling market, with one state-owned operator and around 80–90 private operators, most of which offer online casino, online sports betting, or online card games, and some offer online horse race betting. The legal gambling age is 18. Land-based casino gambling is organized in three state-owned monopoly establishments in the three major cities of Sweden, as well as a number of so called “restaurant casinos,” offering table games with limited stakes.

The present study is a retrospective chart review study on all patients who presented to the gambling disorder treatment unit of Malmö Addiction Center with gambling disorder from January 2017 to August 2021 [12]. An experienced therapist (the third author), familiar with gambling disorder and its treatment, reviewed all medical records and collected information according to a predetermined template. A recent brief paper has reported specifically the self-exclusion data and data describing the breaching of self-exclusion for the eight-month period during 2021, although without more in-depth analyses of patient characteristics (psychiatric comorbidity) and without allowing for the longer time frame since the *Spelpaus*self-exclusion service was introduced [33].

### 2.1. Measures

Information regarding the year of birth, sex, month of first presentation, age of gambling onset, main type of gambling, and comorbidities was extracted, if present from patient medical records. Sex referred to the biological sex (male or female), as indicated by the administrative personal identification number. The types of gambling found and registered were categorized in the following way: online casino (online chance-based games, typically online slot machines, and online casino table games), sports betting (online or land-based), land-based casino gambling (including any type of gambling in legal casino establishments, such as table games), illegal land-based casino gambling, online poker, land-based poker, and slot machine gambling (land-based electronic gambling machines).

For patients presenting from January 2019 onwards, it was also recorded whether a patient had self-excluded and if so, whether they had gambled despite an ongoing self-exclusion. The method of gambling during self-exclusion was also noted. Only new patients, presenting for the first time, were included. Although some patients who presented for the first time in 2018 were still receiving treatment in 2019 and thus had had the opportunity to self-exclude using *Spelpaus*, such information was disregarded when analyzing self-exclusion.

A small number of patients who were referred by mistake to the gambling disorder unit, due to administrative errors, for example, were disregarded. For six patients, the month of first presentation was not available, and they were subsequently removed from the study, such that from 2017 to 2021, 370 patients were included, among whom, 193 had presented for the first time from January 2019 to 2021.

The approximate age was calculated by subtracting the year of birth from the year the patient first presented. For analyses regarding comorbidity, only diagnoses from ICD-10 [33] chapter F (mental and behavioral disorders [34]) were included.

### 2.2. Statistical Methods

Statistical tests were done using IBM SPSS Statistics. Either Mann–Whitney *U* (age) or chi-squared tests (sex, comorbidity, and time periods), for group-wise comparisons, were performed.

### 2.3. Ethics Permission

The study was approved by the Swedish Ethics Review Authority (file number 2021–03636). According to this ethics permission, no informed consent from patients was collected or required for the present type of retrospective analysis.

## 3. Results

### 3.1. Psychiatric Comorbidity in Patients from 2017 to 2021

From the total of 370 patients presenting to Malmö Addiction Center between January 2017 and August 2021, 295 were males and 75 were females. There was no significant trend in the number of patients presenting per half year (*p* = 0.211, linear by linear). There was also no clear trend in comorbidities over time (*p* = 0.739, linear by linear, Table 1).

Comorbidity was more common amongst females than males (*p* < 0.001), with 48% (*n* = 36) of females having other psychiatric diagnoses compared to 25% of males (*n* = 75). The prevalence of diagnoses from ICD-10 block F1 (substance use disorders) did not differ significantly between sexes (*p* = 0.273). Females tended to have more diagnoses from blocks F3 (mood disorders, *p* < 0.001), F4 (neurotic/anxiety-related disorders, *p* < 0.001), and F9 (behavioral/emotional disorders, typically with an onset during childhood, *p* = 0.028). Females were also significantly more likely to have three or more psychiatric diagnoses, i.e., gambling disorder and at least two more diagnoses from different F blocks. In total, the most common group of diagnoses was substance use disorders (F1), as shown in Table 2.

### 3.2. Data regarding Self-Exclusion, 2019–2021

**Figure 1:**
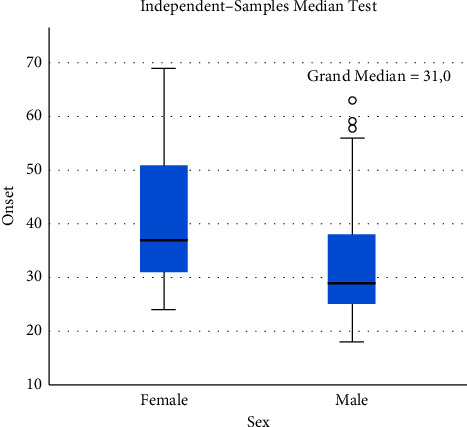
Box-plot diagram showing sex differences in age of onset, with IQR, median, minimum and maximum values, and outliers for patients from January 2019 to August 2021. *N* = 193.

From patients presenting to Malmö Addiction Center from January 2019 onwards (*n* = 193), 151 were males and 42 were females. The median age at first presentation in total was 32 years. Females were generally older at first presentation with a median age of 39 years than males of 30 years (*p* < 0.001, Figure 1). The median age of problem gambling onset was also higher at 30.5 years for females than that of 21 years for males (*n* = 155; *p* < 0.001). For all females for whom information was found, the main type of gambling was online casino. This differed from the males, for whom online casino was also the most popular type of gambling, but who also had a large proportion of sports betting (*p* < 0.001). Information about the main type of gambling was missing in 27 individuals (Table 3).

From 193 patients, 114 reported to have used *Spelpaus* before and six reported they had not. Information was missing in 73 patients. There were 87 male and 27 female self-excluders. Within the group that did not use *Spelpaus*, there were five males and one female. The median age of first presentation was 33.5 years for self-excluders compared to 23.5 years for non-self-excluders (*p* = 0.238). There was no clear trend in the number of patients using *Spelpaus* since 2019 (*p* = 0.771) and also no trend in the proportion of self-excluders that gambled despite self-exclusion (*p* = 0.779, Table 4).

The prevalence of psychiatric comorbidity was 40% in the self-excluders and 17.7% (*p* < 0.001) when considering all patients from 2019 who denied having self-excluded (*n* = 6) or from whom there was no information available on past self-exclusions (*n* = 73).

From 114 self-excluders, 67 reported to have gambled despite self-exclusion; 38 denied gambling despite self-exclusion and information was missing for the remaining nine self-excluders (five females and four males). Out of the 38 who denied gambling despite self-exclusion, 30 were males and 8 were females, and the median age was 34 years. Females were not significantly (*p* = 0.146) more likely to gamble despite self-exclusion (70.4%, *n* = 19) than males, 55.2% (*n* = 48). The median age of first presentation within the group that gambled despite self-exclusion was 30 years.

The proportion of patients with comorbidities was 48% (*n* = 32; 32 patients with no reported comorbidity, three with missing information) in individuals who gambled despite self-exclusion, compared to 34% in those who did not (*n* = 13; *p* = 0.178). The most frequent method to gamble despite self-exclusion was to gamble on unregistered sites (*n* = 40) followed by gambling using another person's ID (*n* = 14); three patients reported gambling via land-based methods and the information was missing in 10 patients.

## 4. Discussion

### 4.1. Clinical Picture in Gambling Disorder Treatment—Sex Differences

The present study showed that females generally began gambling and sought treatment at this specialized treatment unit at an older age than males. This is in accordance with Nower and Blaszczynski [4], who also suggested that the development of severe problematic gambling occurred later in life and also faster in females than in males.

Although online casinos were by far the most common type of gambling in total, a large proportion of males preferred sports betting, while females exclusively gambled in online casinos. This is in agreement with previous research showing that females tend to choose noninteractive and nonstrategic games, such as casinos and slot machines, more frequently than males. The research also showed that males are more prone to choose strategic gambling in a sports context [5]. However, a study on 18–29-year-old Finnish males and females suggested that the preferred types of gambling among females were “weekly lotteries, slot machines, scratch cards, and slow-paced lottery games” [35], whereas the males played online and offline casinos and betting games more often than females. These findings suggest that countries may differ significantly in the preferred types of gambling. The present picture is in line with what has been described from the same treatment unit earlier [12].

Generally, considering all patients since 2017, females more frequently suffered from affective disorders, neurotic, stress-related and somatoform disorders, and behavioral and emotional disorders with onset usually occurring in childhood and adolescence [33]. A study on psychiatric in-patients in Germany and Denmark from 2002 showed that males were overrepresented in F1 diagnoses, whereas females were overrepresented in F3 and F4 diagnoses [36]. The present study also found that females were significantly more likely to have two or more psychiatric diagnoses on top of gambling disorder. Also, interestingly, males were not significantly more likely to suffer from mental and behavioral disorders relating to psychoactive substance use. The latter is in contrast with traditional findings of a more pronounced substance-related comorbidity in males than in females with gambling disorder [37–39], but in more recent findings from the present setting, no significant differences between females and males have been seen in Swedish National Register Data [11] or in online survey data [40]. It goes beyond the scope of the present study to examine the reason behind this finding, from the present and other studies in the same setting. However, it further indicates that mental health comorbidity in many aspects is more severe in females than in males with gambling disorder, such that even substance-related comorbidity does not stand out among male patients.

The findings confirm the impression that other psychiatric disorders may be risk factors for developing gambling disorder; alternatively, that gambling disorder may increase the probability of developing additional psychiatric disorders. It appears that females are particularly vulnerable in this context. Potenza et al. [5] showed that although males and females both experienced high levels of anxiety and depression as a result of gambling disorder, females were more likely to report anxiety and suicide attempts. In this study, information about the temporality of comorbidity onset was not available, and it is therefore beyond the scope of the present study to assess whether gambling disorder was a result of comorbidity or vice versa.

### 4.2. Spelpaus Self-Exclusion

The present study found that out of 120 patients for whom information on the use of *Spelpaus* was found, 114 had chosen to self-exclude (95%) and only six had not. This suggests that there is widespread knowledge of the existence of *Spelpaus* and a positive attitude towards its use in controlling/reducing problematic gambling. The median age of self-excluders was 33.5 years compared to 23.5 years in non-self-excluders. This seems counterintuitive, as one might expect individuals who did not self-exclude to be older patients that preferably gamble offline, for example, in land-based casinos, and may be less aware of the online service *Spelpaus*. It is difficult to speculate on why this might be the case, and more research would be of interest to explore this, in particular in larger study samples.

There was no clear trend over time in the number of patients choosing to self-exclude and those who did not. Simultaneously, the total number of *Spelpaus* users nationwide was rising [20], which suggests that gamblers with a high level of problem severity were aware of and were positive towards *Spelpaus* already soon after its launch and that the use of *Spelpaus* may have spread to gamblers with less severe problems in this time span.

The study also found that psychiatric comorbidity was markedly higher in self-excluders compared to those patients since 2019 who either denied self-excluding or where no information was found regarding self-exclusion (*n* = 73). This suggests that patients with a more severe clinical picture were more likely to resort to *Spelpaus* as a treatment. However, as the comparison group is largely made up of patients for whom information about self-exclusion was not available, the statistical comparison should be interpreted with caution. However, again, individuals who were confirmed to be self-excluded in the data do appear to represent a group with a high degree of problem severity, including psychiatric comorbidity.

### 4.3. Gambling despite Self-Exclusion

As presented, the most common way to gamble despite *Spelpaus* was via unregistered sites. *Spelpaus* only applies to licensed gambling operators, which leave all foreign websites without a Swedish operating license and illegal sites free to access for the self-excluded patient. This is a loophole of a system like *Spelpaus* and it may be a challenge to identify a precise solution to this problem. It is unlikely to be plausible to technically block all foreign gambling websites, and a state-controlled censorship of specific websites would be controversial from a freedom of speech standpoint. The Swedish gambling authority works actively to reduce the number of illegal operators to prevent individuals with gambling problems from continuing gambling, for example, by blocking payments or displaying warnings when a Swedish user accesses an illegal site. A filter individual can choose to apply to electronic devices with Internet access that prevents users from accessing unlicensed gambling websites would be a possible solution. However, such a filter that can accurately detect illegal websites without blocking access to nonviolating websites may be very difficult to attain.

The study also showed that individuals who gambled despite self-exclusion more frequently had psychiatric comorbidities than those that successfully self-excluded from all gambling, suggesting that *Spelpaus* may be more effective in individuals with fewer/no psychiatric comorbidities. Perhaps *Spelpaus* is a tool most effective in gamblers who can use self-exclusion to control/reduce their gambling habits with less severe, nonaddictive gambling patterns. These gamblers might be less likely to seek alternative ways of gambling despite self-exclusion, such as unregistered sites.

As mentioned, close to 80,000 individuals had self-excluded using *Spelpaus* in September 2022 [21]. However, a rough estimate of the current number of individuals with gambling disorder in Sweden would be half that number. Many of the *Spelpaus* users, who control their gambling using *Spelpaus*, might be gamblers with mild/moderate problems. It is possible that this could prevent less severe addictive behaviors from progressing to gambling disorders as present in the patients of this study. It would be of interest to explore this in future research. When interpreting the number of *Spelpaus* users, it is also important to consider that some users might choose to self-exclude in order to receive less advertisement from gambling operators, which are prohibited from sending personalized advertisements, such as emails and text messages, to *Spelpaus* users.

### 4.4. COVID-19

The fact that the turnover for major gambling operators was unchanged or even increased slightly during the pandemic [31] suggests that individuals transitioned from gambling in offline venues or betting on sports to gamble in online casinos. It is interesting to note that in July and August 2020, a time when the World Health Organization was advising countries to implement public health measures such as lockdowns and social distancing guidelines [41], the number of Google searches of the phrase “online casino” reached the highest point recorded to date (records started in 2004) [42]. Research on whether this apparent increase in online gambling might lead to more individuals with problematic gambling or more severe gambling problems would be valuable. The present study showed no significant increase in patients presenting with gambling problems or in patients with psychiatric comorbidities. However, patient presentation may be delayed, considering barriers to seek treatment [10], such that studies covering a longer period of time after the pandemic would be necessary. It is also important to consider that many individuals avoided seeking medical care during the pandemic, which may have reduced a possible increase in the number of patients seeking treatment during that period [43].

## 5. Conclusion

In conclusion, a majority of the patients with gambling disorder, included in this study, had chosen to self-exclude via *Spelpaus*. However, gambling despite self-exclusion was common among these patients. This indicates that there is a general positive attitude towards the possibility of self-exclusion, but that the method needs further development. The reason for the large percentage of patients gambling despite *Spelpaus* could be that it is too easy for self-excluded individuals to circumvent the self-exclusion and gamble in other ways due to the gambling market being too large and easily accessible.

As mentioned, there are no previous studies describing the actual effects of a nation-wide multioperator self-exclusion tool of this kind, likely as it is a relatively new method. Therefore, there is a great need of further studies evaluating *Spelpaus.* The results of this study can hopefully pave the way for future research in this area and also help in further development of self-exclusion sites. It is clear that *Spelpaus* as a self-help tool needs to be developed in order to be able to filter out all forms of gambling platforms, including illegal operators. Policy makers should also keep working on improving gambling regulations to prevent individuals from being able to gamble on unlicensed gambling operators while being self-excluded.

The findings of sex differences in clinical patients in this study show that more research regarding pathological gambling in males than in females need to be done. With the understanding of these differences also comes the possibility to create a better adjusted, personalized treatment. When it comes to comorbidities, the main finding of this study was the confirmed picture that it was significantly more prevalent in females than in males. This highlights that treatment may need to be different for males and females in order to be most effective. These results imply that it is of great importance to recognize the link between gambling disorder and other psychiatric comorbidities in order to identify risk factors and prevent unproblematic gambling.

More studies need to be done in order to obtain more profound information about the effect of *Spelpaus* on individuals with gambling problems. It would be valuable to perform qualitative studies about the patients' own experiences of *Spelpaus*. A study including more information about the patients, for example, data on the severity of the disorder, duration of self-exclusions for each patient, and the exact date of breaching of the patient's *Spelpaus*self-exclusion, could also help to understand the efficacy of self-exclusion in different patient groups. More background information about the socioeconomic situation, living conditions, and lifestyle of the patients could also contribute to a more accurate picture of self-exclusion as a treatment. Future studies on *Spelpaus* may want to include the specific treatments individuals received in addition to self-exclusion.

### 5.1. Study Implications

The present study, carried out in a clinical gambling disorder treatment setting, has a number of clinical implications. First, again, it confirms the elevated rates of psychiatric comorbidity in females with gambling disorder, and therefore, it points to the importance to screen for gambling problems in settings where mental health disorders are treated, and even more so among females with these disorders. Also, the other way around, the detection of poor mental health in individuals with gambling problems proves to be important and can be seen as even more prioritized in females. Also, the study demonstrates that although many individuals who seek treatment for gambling problems have self-excluded from gambling, this cannot be seen as a sufficient intervention in this group with clinical signs of a gambling disorder. Thus, structured treatment and other lifestyle interventions remain of great importance, as it appears that continued gambling despite self-exclusion is common. It underlines the importance of combining self-exclusion programs with structured counselling and referral to evidence-based therapy, instead of offering only the self-exclusion itself. Such a development of a link between the harm-reducing self-exclusion and further therapeutic efforts needs to be addressed by policy makers, clinicians, and in responsible gambling practices of gambling operators. In addition, the high risk of further gambling despite self-exclusion should be acknowledged by other counselling services, enforcement agencies, or debt counsellors who meet clients who have self-excluded from gambling but who remain at risk of further gambling exposure from overseas operators or other unlicensed gambling services.

## 6. Limitations

This study only involved patients from 2017 to 2021 from Malmö Addiction Center in the Skåne Region (in total 370 patients), and therefore, the results may not be representative for a nationwide or international population. With this in consideration, a population sample from another region in Sweden or another country could have given different results than those presented in this study.

The documentation from clinicians left out information regarding a number of patients. Data about age of onset, use of *Spelpaus*, gambling despite *Spelpaus*, comorbidities, and main type of gambling were missing in some patients, which made the groups smaller when certain tests were performed. For example, the sample did only include six persons that did not use *Spelpaus*, which made statistical comparison difficult.

Altogether, the use of health care records presents some limitations, as the documentation is based on information that has been provided during assessment or treatment at the unit. For example, the exact extent of gambling is not available from the documentation used here. For example, the breaching of one's self-exclusion cannot be described more in detail from this type of documentation, such that it cannot be established whether such gambling despite self-exclusion involves a brief breach or a more extended relapse into uncontrolled gambling. Such information would require more in-depth study and should be addressed in future research.

## Figures and Tables

**Table 1:** Proportion of males and females presenting to Malmö Addiction Center and presence of psychiatric comorbidities per half year.

	Total patients	Male	Female^a^	Comorbidity
1st 2017	7	85.7% (6)	14.3% (1)	42.9% (3)
2nd 2017	25	84.0% (21)	16.0% (4)	28.0% (7)
1st 2018	69	84.1% (58)	15.9% (11)	30.4% (21)
2nd 2018	84	76.2% (64)	23.8% (20)	28.6% (24)
1st 2019	34	82.4% (28)	17.6% (6)	29.4% (10)
2nd 2019	36	80.6% (29)	19.4% (7)	41.7% (15)
1st 2020	34	88.2% (30)	11.8% (4)	29.4% (10)
2nd 2020	34	73.5% (25)	26.5% (9)	17.6% (6)
1st 2021	47	72.3% (34)	27.7% (13)	31.9% (15)
Total	370	79.7% (295)	20.3% (75)	30.0% (111)

^a^Linear by linear association = 0.211.

**Table 2:** Prevalence of ICD-10 categories in male and female patients from January 2017 to August 2021.

ICD-10 categories	Total	Male	Female	*p* value
F1	11.6% (43)	12.5% (37)	8.0% (6)	0.273
F3	7.8% (29)	3.4% (10)	25.3% (19)	<0.001
F4	7.2% (36)	7.1% (21)	20.0% (15)	<0.001
F9	4.5% (17)	3.4% (10)	9.3% (7)	0.028
Two or more in addition to gambling disorder	5.6% (21)	3.7% (11)	13.3% (10)	0.001

**Table 3:** Main type of gambling in males, females, and in total, patients from January 2019 to August 2021.

	Male	Female	Total
Online casino	70	39	109
Sports betting	52	0	52
Land-based casino	1	0	1
Online poker	1	0	1
Land-based poker	1	0	1
Slot machine	1	0	1
Unregistered land-based casino	1	0	1
Total	127	39	166

*N* = 166.

**Table 4:** Proportion of patients who self-excluded and the proportion of self-excluders that gambled despite self-exclusion per half year.

	Patients reporting the use of *Spelpaus*^a^	Denied self-exclusion	Self-excluders that gambled despite self-exclusion^b^	Did not gamble despite self-exclusion
1st 2019	61.8% (21)	1	52.4% (11)	6
2nd 2019	55.6% (20)	1	55.0% (11)	9
1st 2020	52.9% (18)	2	56.6% (10)	6
2nd 2020	58.8% (20)	1	60.0% (12)	6
1st 2021	66.0% (31)	1	61.3% (19)	11
Total	59.5% (110)	6	57.3% (63)	38

^a^Linear by linear = 0.771; ^b^linear by linear = 0.779.

## Data Availability

Original data were derived from confidential hospital records and cannot be shared publicly, according to the relevant ethics approval.
